# Ultra-low-power second-order nonlinear optics on a chip

**DOI:** 10.1038/s41467-022-31134-5

**Published:** 2022-08-04

**Authors:** Timothy P. McKenna, Hubert S. Stokowski, Vahid Ansari, Jatadhari Mishra, Marc Jankowski, Christopher J. Sarabalis, Jason F. Herrmann, Carsten Langrock, Martin M. Fejer, Amir H. Safavi-Naeini

**Affiliations:** 1 E.L. Ginzton Laboratory, Stanford University, Stanford, CA 94305 USA; 2 Physics & Informatics Laboratories, NTT Research, Inc., Sunnyvale, CA 94085 USA; 3 Present Address: Physics & Informatics Laboratories, NTT Research, Inc., Sunnyvale, CA 94085 USA; 4 Present Address: Flux Photonics Inc., 580 Crespi Dr Unit R, Pacifica, CA 94044 USA

**Keywords:** Integrated optics, Nonlinear optics

## Abstract

Second-order nonlinear optical processes convert light from one wavelength to another and generate quantum entanglement. Creating chip-scale devices to efficiently control these interactions greatly increases the reach of photonics. Existing silicon-based photonic circuits utilize the third-order optical nonlinearity, but an analogous integrated platform for second-order nonlinear optics remains an outstanding challenge. Here we demonstrate efficient frequency doubling and parametric oscillation with a threshold of tens of micro-watts in an integrated thin-film lithium niobate photonic circuit. We achieve degenerate and non-degenerate operation of the parametric oscillator at room temperature and tune its emission over one terahertz by varying the pump frequency by hundreds of megahertz. Finally, we observe cascaded second-order processes that result in parametric oscillation. These resonant second-order nonlinear circuits will form a crucial part of the emerging nonlinear and quantum photonics platforms.

## Abstract (web summary)

Here, the authors demonstrate a chip-scale device that realizes a comprehensive set of resonant second order nonlinear processes including optical parametric oscillation with a threshold power of 70 microwatts.

## Introduction

The remarkable progress and impact of silicon photonics has led to the development of complex and high-performance optical systems for communications, sensing, and quantum and classical information processing. In addition to linear passives, modulators, and detectors, many applications would significantly benefit from versatile nonlinearities. Integrated photonic circuits made with centrosymmetric silicon^1,2^ or amorphous silicon nitride^3–5^ confine light in dispersion-engineered waveguides and resonators to enhance the third-order optical nonlinearity and have been used successfully to demonstrate optical frequency combs^6–8^, wavelength conversion^9^, and squeezed light generation^10,11^. Efforts continue to further improve the efficiency and tailorability of these devices by incorporating second-order nonlinearity to enable stronger interactions at lower power and reduce the number of competing nonlinear processes that emerge. Second-order nonlinearity can be introduced by breaking the symmetry of a crystal^12,13^ or heterogeneously integrating a non-centrosymmetric material^14,15^.

**Fig. 1:**
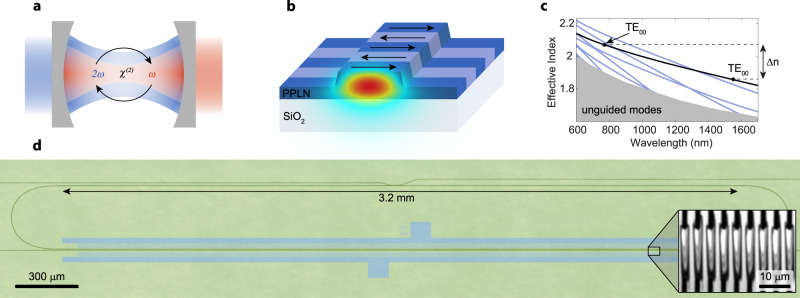
Integrated, resonant second-order nonlinear optical device. **a** Schematic of a resonant second-order nonlinear optical device. Driving the cavity with second harmonic light (blue) results in optical parametric oscillation at the fundamental; Driving at the fundamental frequency generates second harmonic light. **b** Periodically-poled lithium niobate ridge waveguide that confines light to a small volume and supports nonlinear interactions. The transverse electric field parallel to the surface of the chip is plotted for the fundamental spatial mode. **c** Effective index of the waveguide spatial modes as a function of wavelength. Periodic poling compensates for the phase velocity mismatch (∝Δ*n*) between fundamental and SH modes. **d** The racetrack resonator used as a platform for nonlinear optics. Laser light is injected through an evanescent coupler on the top and undergoes nonlinear interaction in the bottom, periodically-poled section. The resonator is not coupled to the waveguide below it, which serves as a tool for poling diagnostics. The laser confocal microscope picture has been colorized; blue shading highlights the poling electrodes location during the fabrication process. Inset shows a second-harmonic microscope picture of the poled region. Inverted domains stretch between black electrode fingers.

Alternatively, photonic circuits may be built directly from a *χ*^(2)^ nonlinear material such as aluminum nitride^16^ or lithium niobate (LN). LN can be periodically poled to compensate for phase mismatch due to dispersion^17–23^ and supports high-*Q* optical resonances^24^, a large electro-optic coefficient^25–27^, and Kerr nonlinearity^28,29^. Here we show ultra-efficient resonant *χ*^(2)^ nonlinear optical functions (Fig. 1a) on a chip that incorporates quasi-phase-matching with a nonlinear optical resonator. We overcome parasitic effects that so far have limited the stability and performance of integrated LN devices to demonstrate second-order processes such as optical parametric oscillation, which have previously only been observed in LN bulk resonators^30,31^. We operate an optical parametric oscillator (OPO) across degenerate and non-degenerate regimes and show tuning of the emission spectrum across one THz by adjusting the frequency of the pump across hundreds of MHz, all at room temperature. We also show frequency doubling that leads to highly-enhanced effective third-order nonlinearity, resulting in cascaded parametric oscillation. The presented coupled-mode theory accurately models the dynamics and confirms the operating modes of the device.

In this work, we make waveguides from a thin film of X-cut lithium niobate (Fig. 1b), which has its largest electro-optic and *χ*^(2)^ tensor components parallel to the surface of the chip. This orientation has been used in recent demonstrations of telecommunications modulators^25,27^, frequency combs^26^, cryogenic frequency converters^32–34^, and sources exhibiting quantum correlations^35,36^, which form an emerging thin-film LN platform. We use magnesium oxide (MgO) doped lithium niobate to suppress pump-induced absorption and reduce the photorefractive damage typically experienced by devices fabricated with undoped congruently grown lithium niobate^37^.

## Results

Due to both its geometry and material properties, the dispersion of the waveguide introduces a phase velocity mismatch proportional to Δ*n*—the difference in refractive indices between fundamental (FH) and second harmonic (SH) modes as shown in Fig. 1c. To achieve efficient nonlinear interactions, we compensate for the phase velocity mismatch by periodically poling the LN crystal. This quasi-phase-matching technique provides momentum conservation and enables the use of the same fundamental transverse electric (TE) spatial mode at both wavelengths^19,20^. These modes exhibit the tightest confinement and have the strongest overlap with the large *d*_33_ component of the *χ*^(2)^ nonlinear tensor, thereby enabling a large nonlinear interaction rate. We use a poling period of Λ = *λ*_SH_/Δ*n* ≈ 3.7 μm. The inset of Fig. 1d shows a second-harmonic microscope picture of the periodic poling before waveguide fabrication. We observe the formation of oblong shapes with greyscale fringes between finger electrodes (black) that correspond to inverted crystal domains^38^.

The waveguide forms a racetrack resonator with a straight section length *L* of 3.2 mm (see Fig. 1d) that supports resonances across a broad range of wavelengths. We employ a phase-mismatched waveguide coupler design to efficiently couple light into the resonator at both FH and SH frequencies^39^. Near the FH and SH frequencies, we measure intrinsic quality factors exceeding 10^6^, which dramatically enhance nonlinear processes by increasing the lifetimes of the interacting photons.

The resonances around the fundamental and second harmonic bands have frequencies *ω*_*m*_ and Ω_*k*_, with corresponding linewidths *κ*_*A*,*m*_ and *κ*_*B*,*k*_. We drive with pump frequency nearest to *ω*_0_ and Ω_0_ in the following experiments. The FH mode frequencies vary with index as *ω*_*m*_ ≈ *ω*_0_ + *ζ*_1_*m* + *ζ*_2_*m*^2^/2, where *ζ*_1_ is the free spectral range and *ζ*_2_ is a dispersion parameter. Temperature tuning of the devices changes the relative detuning between the modes and gives us fine control over the modal detuning *μ* ≡ Ω_0_ − 2*ω*_0_. The small free spectral range of our device (17.26 GHz and 16.45 GHz at the FH and SH, respectively), allows us to tune *μ* while keeping the device within a few degrees of room temperature.

The *χ*^(2)^ optical nonlinearity of the material causes two FH resonances at *ω*_*m*_ and *ω*_*n*_, and the SH resonance at Ω_*k*_ to interact with each other at a rate *g*_*k*,*nm*_. All of the dynamics of this system are captured by a set of coupled-mode equations for the fundamental (*A*_*m*_) and second harmonic (*B*_*k*_) field amplitudes. These amplitudes correspond to intracavity energies *ℏω*_*m*_∣*A*_*m*_∣^2^ and *ℏ*Ω_*k*_∣*B*_*k*_∣^2^, and evolve in time as

1 $$\frac{{{{{{{{\rm{d}}}}}}}}}{{{{{{{{\rm{d}}}}}}}}t}{A}_{m}=-\frac{{\kappa }_{A,m}}{2}{A}_{m}-2i\mathop{\sum}\limits_{kn}{g}_{k,nm}{A}_{n}^{* }{B}_{k}{e}^{-i{\delta }_{k,nm}t}$$

2 $$\frac{{{{{{{{\rm{d}}}}}}}}}{{{{{{{{\rm{d}}}}}}}}t}{B}_{k}=-\frac{{\kappa }_{B,k}}{2}{B}_{k}-i\mathop{\sum}\limits_{mn}{g}_{k,nm}^{* }{A}_{m}{A}_{n}{e}^{+i{\delta }_{k,nm}t},$$

with *δ*_*k*,*nm*_ ≡ Ω_*k*_ − *ω*_*n*_ − *ω*_*m*_. To operate as an optical parametric oscillator (OPO), a laser driving term is added to the first equation, while adding a laser driving term to the second equation causes second harmonic generation (SHG) and eventually operation as a cascaded OPO.

Optical parametric oscillation occurs when the second-harmonic mode is driven to a sufficiently large steady-state cavity occupation ∣*B*_0_∣^2^. The system will begin to oscillate at this input power, either as a degenerate OPO with emission into *ω*_0_ mode or as a nondegenerate OPO emitting into a pair of modes *ω*_±*m*_. The mode of oscillation is that with the lowest threshold *P*_th,*m*_, which strongly depends on laser detuning Δ, modal detuning *μ*, total loss *κ*, extrinsic loss *κ*^(e)^, and dispersion *ζ*_2_*m*^2^:

3 $${P}_{{{{{{{{\rm{th}}}}}}}},m}=	\frac{\hslash {{{\Omega }}}_{0}}{16| {g}_{0,-mm}{| }^{2}}\frac{1}{{\kappa }_{B,0}^{{{{{{{{\rm{(e)}}}}}}}}}}\left({{{\Delta }}}^{2}+{\left({\kappa }_{B,0}/2\right)}^{2}\right)\\ 	\times \left({\left({{\Delta }}+\mu -{\zeta }_{2}{m}^{2}\right)}^{2}+{\kappa }_{A,m}{\kappa }_{A,-m}\right).$$

The pair of modes *ω*_±*m*_ with the lowest loss rates will experience the lowest threshold and oscillate first as we increase the pump power. Above the threshold, the OPO output power follows a square-root function of the input power *P*_*B*,0_ provided that the input power is not sufficiently large to produce simultaneous oscillation of multiple mode pairs:

4 $${P}_{{{{{{{{\rm{out}}}}}}}}}=\frac{4{\eta }_{B,0}}{{{{\Omega }}}_{0}}\left({\eta }_{A,m}{\omega }_{m}+{\eta }_{A,-m}{\omega }_{-m}\right)\times {P}_{{{{{{{{\rm{th,m}}}}}}}}}\left(\sqrt{\frac{{P}_{B,0}}{{P}_{{{{{{{{\rm{th,m}}}}}}}}}}}-1\right).$$

Here ${\eta }_{k,j}\equiv {\kappa }_{k,j}^{{{{{{{{\rm{(e)}}}}}}}}}/{\kappa }_{k,j}$ is the cavity-waveguide coupling efficiency for *k* ∈ {*A*, *B*} and *j* being the index of a specific mode.

Driving the fundamental frequency *ω*_0_ generates light at the second harmonic mode Ω_0_. The efficiency of this process has a linear dependence on input power in the low power regime. Once the additional nonlinear conversion loss experienced by the FH mode (proportional to 8∣*g*_0,00_*A*_0_∣^2^/*κ*_*B*,0_ with zero detuning) approaches the cavity linewidth *κ*_*A*,0_, the cavity’s effective coupling efficiency to the input light is reduced. This leads to a sub-linear *P*^−1/3^ dependence as the process now converts a substantial amount of pump photons to second harmonic photons in the resonator. A competing oscillation instability leading to parametric oscillations may prevent observing this power law.

At high FH pump powers, the intracavity SH photon population at Ω_0_ is large enough to create an instability in the field amplitude of FH modes *A*_*m*_, causing parametric oscillations when the generated SH intracavity photon number exceeds the threshold condition:

5 $${\left|{B}_{0}\right|}^{2}\ge \frac{1}{16{\left|{g}_{0,-mm}\right|}^{2}}\left({\left(2\delta +\mu -{\zeta }_{2}{m}^{2}\right)}^{2}+{\kappa }_{A,m}{\kappa }_{A,-m}\right).$$

We call this a cascaded OPO, since a cascade of two back-to-back *χ*^(2)^ processes leads to parametric oscillation. The threshold for a cascaded OPO is a function of pump detuning *δ*, modal detuning *μ*, and dispersion *ζ*_2_*m*^2^.

**Fig. 2:**
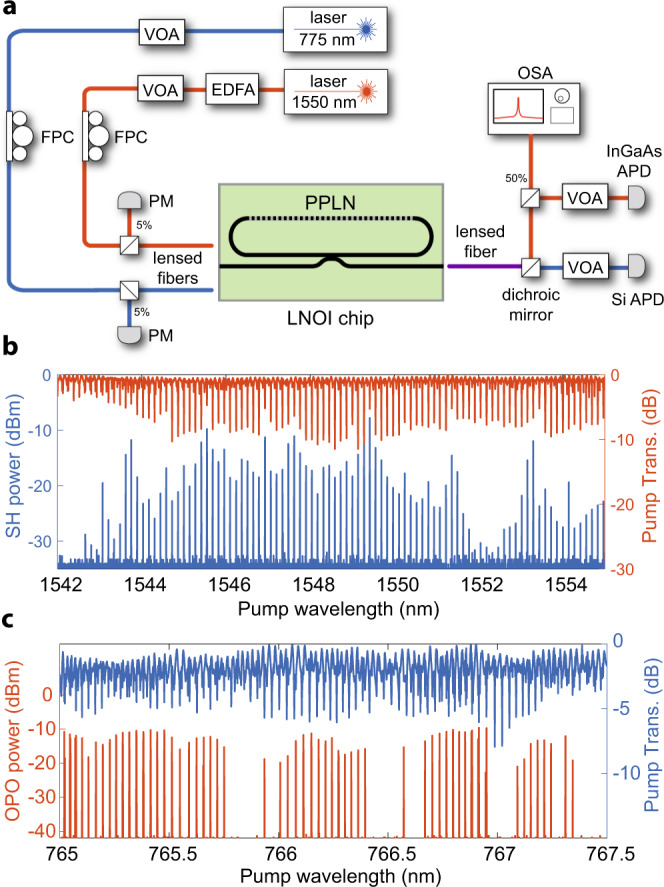
Characterization of nonlinear devices. **a** Experimental setup. We couple light at around 775 or 1550 nm wavelengths onto the chip by aligning a lensed fiber to a cleaved edge facet to excite the coupling waveguide. Light is outcoupled from the chip and demultiplexed to detect the fundamental and second harmonic light separately. **b** Scanning the near-infrared laser shows that second-harmonic generation occurs at wavelengths corresponding to modes of the resonator. **c** Scanning the blue pump laser across wavelength shows that many resonances surpass the parametric oscillation threshold. VOA variable optical attenuator, EDFA erbium-doped fiber amplifier, FPC fiber polarization controller, PM power meter, OSA optical spectrum analyzer, APD avalanche photodiode.

We experimentally probe the nonlinear devices with the setup presented in Fig. 2a; we use two input paths to drive the resonator with fundamental and second harmonic frequency light—shown in red and blue, respectively. We use the path connected to a tunable laser operating in telecommunication wavelengths to study the SHG and cascaded parametric oscillation processes. To drive a direct OPO, we use the input path connected to the shorter wavelength laser. The light is coupled into and out of the chip using lensed fibers. We separate the output light using a free-space setup with a dichroic mirror and send it to Si and InGaAs avalanche photodiodes. We show examples of transmission spectra and corresponding SHG and OPO signals in Fig. 2b and c, respectively. We expect to see variation in the efficiency of second-order processes when probing multiple resonances due to changes in the modal detuning *μ* across the band for a fixed temperature due to the FSR mismatch between FH and SH and variations of the quality factors of participating modes. For spectrally-resolved measurements, we send part of the FH light to the optical spectrum analyzer. We calibrate the fiber-to-chip coupling efficiency based on power transmission measurements and fits of theoretical models to nonlinear response data (see “Methods”). The typical edge coupling efficiency across devices on the chip is 25–40% at telecom wavelengths and 10–20% for the second harmonic depending on fibers and alignment. For each experiment, we measure these efficiencies to within less than a percent uncertainty (see Table 1 in the "Methods" section). All of the presented data refers to the on-chip power, accounting for the edge coupling loss.

**Table 1:** Summary of the measured device parameters.

Device	*λ*_*A*_ (nm)	*λ*_*B*_ (nm)	*Q*_*A*_ (10^6^)	${Q}_{A}^{{{{{{{{\rm{(i)}}}}}}}}}\,(1{0}^{6})$	*Q*_*B*_ (10^6^)	${Q}_{B}^{{{{{{{{\rm{(i)}}}}}}}}}\,(1{0}^{6})$	*g*_0,*nm*_ (kHz)	*η*_FH_ (%)	*η*_SH_ (%)
OPO	$$\begin{array}{l}{\lambda }_{A,m}=1521.05\\ {\lambda }_{A,-m}=1542.43\end{array}$$	765.77	$$\begin{array}{l}{Q}_{A,m}=0.68\\ {Q}_{A,-m}=0.94\end{array}$$	$$\begin{array}{l}{Q}_{A,m}^{{{{{{{{\rm{(i)}}}}}}}}}=0.80\\ {Q}_{A,-m}^{{{{{{{{\rm{(i)}}}}}}}}}=1.50\end{array}$$	0.88	1.50	150	37	13
SHG	1549.40	774.70	0.74	1.2	0.82	1.2	130	26	11

We summarize the wavelengths (*λ*_*A*_ and *λ*_*B*_), total (*Q*), and internal (*Q*^(i)^) quality factors of all of the resonances used in our OPO and SHG experiments. We list the nonlinear coupling factors (*g*_0,*nm*_) and edge coupling efficiencies at both wavelengths (*η*_FH_ and *η*_SH_) for particular devices.

**Fig. 3:**
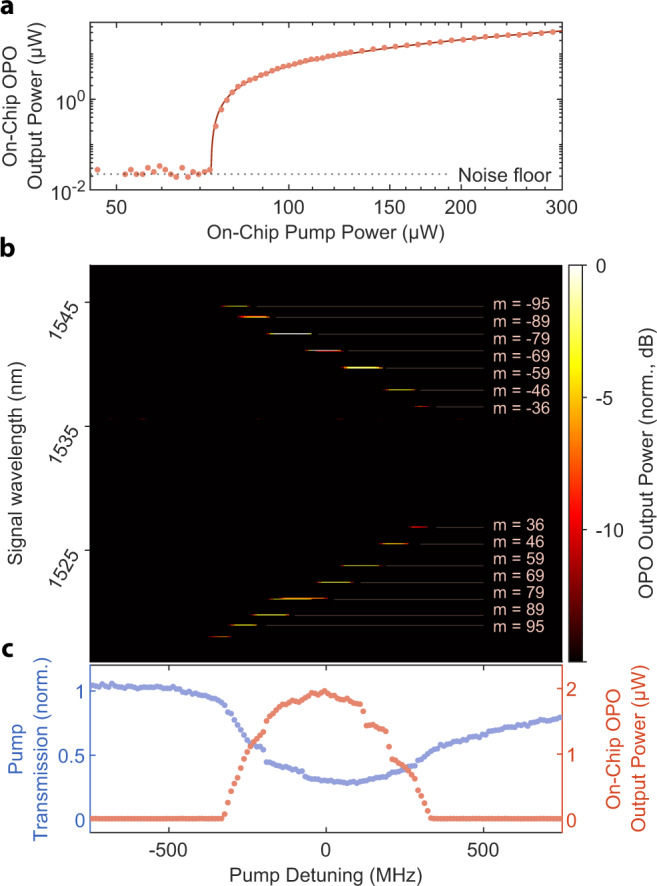
Optical parametric oscillation. **a** Threshold characterization of the OPO, we increase the 765.8 nm laser power until the oscillation begins at a threshold of around 73 μW; above it, the OPO power output follows a square-root relation. Dotted line represents detection noise floor. **b** Tuning of non-degenerate OPO emission with pump wavelength, at a pump power of 250 μW. We can select OPO signal/idler pairs spanning 1537–1545 nm (1527–1519 nm) as the pump is swept over 650 MHz. **c** SH resonance lineshape (blue points) aligned with the OPO response (red points) collected above threshold (250 μW) shows steps corresponding to switching between signal/idler pairs.

We study the OPO by driving the device at around 765.8 nm and recording the generated light at close to twice the wavelength. We temperature tune the modal detuning *μ* close to zero to achieve degenerate operation (see Fig. 5 in the "Methods" section). Given the modal detuning’s temperature dependence and our device’s comparatively small free-spectral range (about 17 GHz), we achieve an optimal operating point close to the room temperature, at 25.65 ^∘^C. For the threshold measurement we detune *μ* from zero to allow the most efficient pair of modes at *ω*_±*m*_ to oscillate, following Eq. (3) (see also the condition defined by Eq. (16) in the “Methods” section). We plot the power of the generated near infrared light in Fig. 3a. The output power vs. input power curve reveals the threshold of oscillation around 73 μW, which we extract from fitting Eq. (4). A maximum efficiency of 11% is measured. Tuning the pump laser wavelength allows for effective selection for the frequencies of oscillating signal-idler pairs of modes. By changing the laser detuning Δ, we observe seven different OPO wavelength pairs generated in the resonator. Figure 3b shows the OPO emission spectrum as a function of pump detuning with a pump power of 250 μW. By tuning the pump laser by just 650 MHz, we can address signal modes across a band of over 1 THz. Figure 3c shows the pump transmission and OPO emitted power as a function of the pump detuning. Detunings of the pump laser relative to the SH cavity mode result in exciting different OPO modes. We can resolve steps on the transmission and OPO emitted power that correspond to switching between different operation modes.

**Fig. 4:**
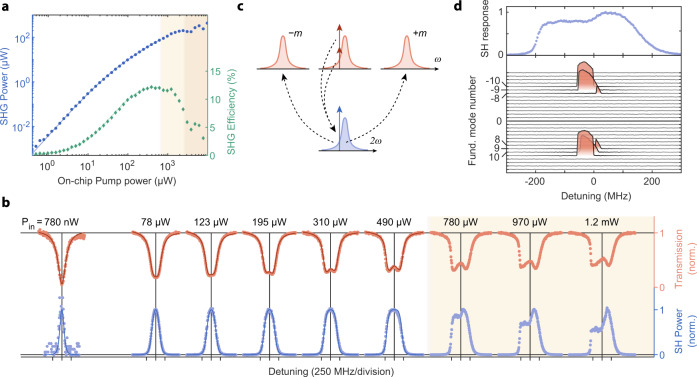
Second harmonic generation and cascaded OPO. **a** 774.7 nm output power as a function of 1549.4 nm input power (blue circles, left axis) and SHG efficiency (green diamonds, right axis). Solid lines represent theoretical prediction. Light yellow shading corresponds to the region where we observe cascaded OPO. Darker shaded region have competing third-order nonlinear processes. **b** Transmission (top) and SH (bottom) lineshapes evolve as a function of power. We plot theoretical curves (solid lines) on the top of data (red and blue points) up to the limit where the two-mode model breaks down and results in a cascaded OPO. A single parameter, the modal detuning *μ*, is varied by about 0.04*κ* between fits. **c** Cascaded OPO scheme—photons at the fundamental frequency drive the SHG process and create light at 2*ω*. Sufficiently high power of the SH can drive the parametric oscillation back in the fundamental frequency range. **d** Measured cascaded OPO, we observe light generation in modes symmetrically spaced from the pump frequency as the SH develops asymmetric lineshape shape (top panel).

To demonstrate second harmonic generation, we drive the FH mode at 1549.4 nm and measure the resulting frequency doubled light at the output. The device temperature is 30.5 ^∘^C. Figure 4a shows the peak SH power generated as a function of input FH power. A maximum efficiency of 12% is achieved with 390 μW of input power in the feed waveguide, which agrees with the coupled-mode theory (solid lines) that includes only the *A*_0_ and *B*_0_ fields. Figure 4b shows how the transmission lineshape and the SH response change as a function of pump power. As the pump power increases, the transmission lineshape widens and becomes shallower due to the additional two photon loss induced by the nonlinearity. At pump powers around 200 μW, the transmission lineshape forms two distinct valleys, consistent with our coupled-mode theory simulations.

**Fig. 5:**
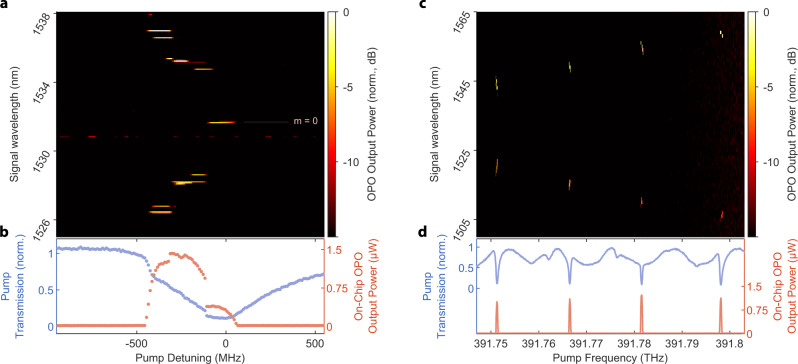
Tuning between degenerate and nondegenerate optical parametric oscillation. **a** Tuning of the OPO close to *μ* = 0, at a pump power of 250 μW. We observe degenerate parametric oscillation at 1531.7 nm (*m* = 0) and nondegenerate operation for blue-detuning of the pump laser. **b** SH resonance lineshape (blue points) aligned with the OPO response (red points) collected above threshold with 250 μW of on-chip pump power. The distinct feature at zero-detuning corresponds to degenerate oscillation. **c** Sweeping the pump laser over four neighboring modes shows that the signal/idler pair center frequencies are different for each OPO. This is due to the different modal detuning *μ* = 0 experienced by each OPO due to the difference in dispersion at 765 and 1530 nm. The higher frequency OPOs have larger ∣*μ*∣ (see discussion in the section “Parametric oscillation theory”). This shows that additional tuning range of the device's output frequency can be extended to about 2.75 THz by utilizing multiple OPOs of a single resonator while keeping the chip temperature fixed. **d** SH resonance lineshape (blue line) aligned with the OPO response (red line) collected above threshold at 250 μW of pump power on chip.

At higher input powers (the yellow shaded region of Fig. 4b), the SH response becomes asymmetrical with a distinct drop in SH power for negative pump detunings, *δ* < 0. At these powers, the intracavity SH light is intense enough to create an instability in the field amplitude of the fundamental modes at *ω*_±*m*_, causing parametric oscillations as visualized in Fig. 4c. The cascade of two *χ*^(2)^ processes creates the parametric oscillation. The normal dispersion of the waveguide (*ζ*_2_ < 0) creates a lower threshold condition for negative pump detunings (*δ* < 0), see Eq. (5). The drop in SH output power at those laser detunings is because SH light at Ω_0_ converts back to FH power at *ω*_±*m*_.

We spectrally resolve the cascaded parametric oscillations as a function of laser detuning and confirm that the first sideband fundamental modes oscillate at a threshold of 690 μW of on-chip pump power. Figure 4d shows multiple sideband oscillations that occur at a pump power of 930 μW. Particular signal-idler pairs oscillate as a function of pump laser detuning as expected from Eq. (5). Disorder in the mode spacing and quality factors causes certain mode pairs to oscillate before others, consistent with coupled-mode simulations.

## Discussion

We expect to find ultra-efficient second-order nonlinear photonic circuits, such as the frequency doubler and parametric oscillator demonstrated in this work, in a number of emerging low-power and quantum applications in the near future. Together with the high-performance integrated devices and components that are being developed for the thin-film LN platform, the promise of a new class of versatile integrated photonic technologies may soon be realized. In addition to sources of broadband and quantum light for sensing and communications, integrated ultra-low-power OPOs can be used for computation with coherent Ising machines^40^ and cluster states^41,42^. *Note:* In the final stages of preparing this manuscript we became aware of a demonstration of low-power optical parametric oscillator in a lithium niobate microresonator^43^.

## Methods

### Fabrication

We fabricate all devices with X-cut thin-film lithium niobate-on-insulator (LNOI) wafers. The material consists of a 500 nm film of LN bonded to a 2 μm layer of silicon dioxide on top of an LN handle wafer.

We pattern the optical devices using electron beam lithography (JEOL 6300-FS, 100-kV) and transfer the design to the LN via Argon ion milling with Ar flow of 15 sccm and a 750 V accelerating voltage. The waveguide width is 1.2 μm, and the etch depth is 300 nm which leaves a 200 nm slab of LN beneath the waveguide. We deposit 700 nm of PECVD silicon dioxide at a temperature of 350 ^∘^C as cladding.

We perform the periodic poling step before waveguide fabrication. For periodic poling, we use electron-beam evaporated Cr electrodes with an electron beam lithography-based liftoff process and apply high-voltage pulses similar to Nagy et al.^44^ to invert the crystal domains. Upon completion of the poling, we remove the electrodes. We only pole one side of the racetrack resonator, but, in principle, both sections could be quasi-phase-matched and increase the nonlinear coupling rate. This design would require careful control of the relative phase of the fundamental and second harmonic light. The waveguide we used has normal dispersion; as a result, the bandwidth of the quasi-phase-matching was about 10 nm.

Chip edge facet preparation is done using a DISCO DFL7340 laser saw. High energy pulses are focused into the substrate to create a periodic array of damage locations, which act as nucleation sites for crack propagation and result in a uniform and smooth cleave.

### Experimental setup

We characterize fabricated devices in a simplified experimental setup shown in Fig. 2a. In the FH input path, we use SMF-28 fibers. 5% of the laser light (Santec TSL-550, 1480–1630 nm) goes into a Mach-Zehnder interferometer (MZI) with an FSR of 67.7  MHz used to calibrate the relative wavelength during laser wavelength sweeps (not shown in Fig. 2a). 95% of the light goes to erbium-doped fiber amplifier (EDFA) with a fixed output power of 250 mW followed by a variable optical attenuator. Next, the light passes through a fiber polarization controller (FPC), and we tap 5% of it just before the input lensed fiber for power calibration with a power meter (Newport 918D-IR-OD3R). The light then couples to the chip facet through an SMF-28 lensed fiber.

In the SH path, we use a Velocity TLB-6700 laser that operates in the 765–781 nm range. This entire path uses 780HP fiber to maintain single-mode operation. A 5% tap outcouples part of the light an MZI with an FSR of 39.9 MHz to calibrate laser wavelength sweeps (not shown in Fig. 2a). A variable optical attenuator controls the remaining laser power, and we control the polarization with an FPC. 5% of the light goes to a power meter (Newport 918D-SL-OD3R) for input power calibration, and we focus the rest of it on the chip facet through a 780HP lensed fiber.

Once the light exits the output edge facet of the chip, we collect it into a lensed SMF-28 fiber, similar to the one used in the FH input path. We outcouple the light into free space and demultiplex with a 1000 nm short pass dichroic mirror. After the dichroic mirror, SH and FH paths are additionally filtered to ensure no cross talk, and we detect SH and FH light with avalanche photodiodes (Thorlabs APD410A and Thorlabs APD410, respectively). Variable optical attenuators are used before the APDs to avoid saturation. We split 50% of the FH light into an optical spectrum analyzer (OSA, Yokogawa AQ6370D) for spectrally-resolved measurements of the OPO.

We use different, but similar, devices on the same chip for the SHG and OPO experiments. The chip sits directly on a thermo-electric cooler for temperature adjustment.

### OPO characterization

We characterize all of the optical resonances that take part in the optical parametric oscillation using linear spectroscopy at powers substantially below nonlinear effects. For these measurements, we sweep the wavelength of tunable lasers in the FH and SH bands and fit the transmission dips with lorentzian lineshapes. We determine the total and intrinsic quality factors of the second harmonic mode to be *Q*_*B*,0_ = 0.88 × 10^6^ and ${Q}_{B,0}^{{{{{{{{\rm{(i)}}}}}}}}}=1.5\times 1{0}^{6}$, respectively. We find the quality factors of the OPO signal modes corresponding to the curve in Fig. 2a to be: *Q*_*A*,*m*_ = 0.68 × 10^6^, ${Q}_{A,m}^{{{{{{{{\rm{(i)}}}}}}}}}=0.8\times 1{0}^{6}$, *Q*_*A*,−*m*_ = 0.94 × 10^6^, ${Q}_{A,-m}^{{{{{{{{\rm{(i)}}}}}}}}}=1.5\times 1{0}^{6}$. We perform an independent second harmonic generation measurements to determine if the FH and SH modes are under or overcoupled. The analysis of transmission lineshapes as a function of pump power confirms that all modes are undercoupled. From the determined threshold of 73 μW we deduce a coupling rate ∣*g*_0,−*mm*_∣ of 150 kHz which is close to the simulated value of 186 kHz.

We measure the input fiber-to-chip coupling with an independent transmission measurement using 780HP lensed fibers at the input and the output chip edges. We assume the input and output coupling is identical, an assumption based on experience with multiple devices on the chip used for the experiment, and find the input edge coupling efficiency to be 13%. We extract the output fiber-to-chip coupling efficiency at the OPO wavelength by fitting the data in Fig. 3a to *η*_FH_*P*_out_ using Eq. (4). We infer *η*_*FH*_ = 37% coupling efficiency, which we confirm with an independent transmission measurement.

### SHG characterization

We characterize the modes contributing to the second harmonic generation in an analogous way to the OPO. From the Lorentzian fits at low power we find quality factors of *Q*_*B*,0_ = 0.82 × 10^6^, ${Q}_{B,0}^{{{{{{{{\rm{(i)}}}}}}}}}=1.2\times 1{0}^{6}$, *Q*_*A*,0_ = 0.75 × 10^6^, and ${Q}_{A,0}^{{{{{{{{\rm{(i)}}}}}}}}}=1.2\times 1{0}^{6}$. Moreover, we use a method for fitting nonlinear lineshapes at high power, as mentioned in the main text. For this purpose, we solve Eqs. (31) and (30) numerically and fit the resulting curves as a function of detuning to the data. We use the ten lineshapes at the pump power between 80 and 620 μW, which allows us to observe changes due to the second-order nonlinearities but avoid the effects of the cascaded OPO. From this procedure we find average *Q*_*A*,0_ = 0.74 × 10^6^, and ${Q}_{A,0}^{{{{{{{{\rm{(i)}}}}}}}}}=1.2\times 1{0}^{6}$ and standard deviation of <4% which agrees with the low power fit. From fitting nonlinear lineshapes, we also extract the coupling rate ∣*g*_0,00_∣ to be about 130 kHz, which agrees with our theoretical prediction of 170 kHz. In the main text, we use averaged values to plot the theoretical lineshapes and only vary the modal detuning to account for small temperature fluctuations. For the SHG device, we make transmission measurements and find the coupling efficiencies to be to be 26% and 11% at the FH and SH, respectively.

We calculate the theoretical relationship between the pump power, SHG power, and SHG efficiency by numerically solving Eqs. (31) and (30) for zero detuning. For the solid lines plotted in Fig. 4a, we use quality factors and the nonlinear coupling rate from the measurements described in the previous paragraph.

### Resolving OPO lines

We use an OSA (Yokogawa AQ370D) to characterize the frequency content of the OPO output spectrum as a function of pump laser detuning. With a constant pump power, we repeatedly sweep the laser wavelength across the SH resonance and record the SH and FH response with APDs (see section “Experimental setup”). A portion of the generated FH light is detected by the OSA operating in zero-span mode with a 0.1 nm filter bandwidth, which is less than the ~0.135 nm free spectral range of the FH modes. We step the center wavelength of the OSA across a 40 nm span with a 50% overlap in OSA filter spans. We record the detected power on the OSA synchronously with the APD detector voltages for each wavelength step. Repeated laser sweeps with different OSA filter center wavelengths produce a map of the OPO frequency content as a function of laser detuning shown in Fig. 3b.

To characterize the cascaded parametric oscillations as shown in Fig. 5d, we first find every potential OPO line’s precise location (*ω*_*m*_) by performing a broad sweep of the FH pump laser and record the resonance frequencies. We then proceeded with the measurement in an identical fashion to the standard OPO, but with the 0.1 nm wide OSA filters placed precisely at the FH mode locations without any overlap between filters.

### Coupled mode theory equations

The Hamiltonian of the system is used to find the equations of motion in the rotating frame.

6 $$\frac{{{{{{{{\rm{d}}}}}}}}}{{{{{{{{\rm{d}}}}}}}}t}{A}_{m}=-\frac{{\kappa }_{A,m}}{2}{A}_{m}-2i\mathop{\sum}\limits_{kn}{g}_{k,nm}{A}_{n}^{* }{B}_{k}{e}^{-i{\delta }_{k,nm}t}-\sqrt{{\kappa }_{A,m}^{{{{{{{{\rm{(e)}}}}}}}}}}{F}_{m}{e}^{-i({\omega }_{L}-{\omega }_{m})t}$$

7 $$\frac{{{{{{{{\rm{d}}}}}}}}}{{{{{{{{\rm{d}}}}}}}}t}{B}_{k}=-\frac{{\kappa }_{B,k}}{2}{B}_{k}-i\mathop{\sum}\limits_{mn}{g}_{k,nm}^{* }{A}_{m}{A}_{n}{e}^{+i{\delta }_{k,nm}t}-\sqrt{{\kappa }_{B,k}^{{{{{{{{\rm{(e)}}}}}}}}}}{G}_{k}{e}^{-i({{{\Omega }}}_{L}-{{{\Omega }}}_{k})t},$$

with *δ*_*k*,*nm*_ = Ω_*k*_ − *ω*_*n*_ − *ω*_*m*_. *A*_*m*_ is the fundamental field amplitude at *ω*_*m*_, and *B*_*k*_ is the second harmonic field amplitude at Ω_*k*_.

### Parametric oscillation theory

We consider the case where the SH modes are driven at frequency Ω_*L*_ and the *A* modes are not excited and calculate the stability criterion for the *A* modes based on Eq. (7):

8 $$\frac{{{{{{{{\rm{d}}}}}}}}}{{{{{{{{\rm{d}}}}}}}}t}{B}_{0}=-\frac{{\kappa }_{B,0}}{2}{B}_{0}-\sqrt{{\kappa }_{B,0}^{{{{{{{{\rm{(e)}}}}}}}}}}{G}_{0}{e}^{-i({{{\Omega }}}_{L}-{{{\Omega }}}_{0})t}.$$

We go into a rotating frame ${B}_{0}={\tilde{B}}_{0}{e}^{-i{{\Delta }}t}$ with frequency Δ = Ω_*L*_ − Ω_0_ defined as the detuning between the laser drive and the *B* mode, which we can solve in steady-state to obtain:

9 $${\tilde{B}}_{0}=\frac{\sqrt{{\kappa }_{B,0}^{{{{{{{{\rm{(e)}}}}}}}}}}{G}_{0}}{i{{\Delta }}-{\kappa }_{B,0}/2}.$$

We now consider two *A* modes at frequencies *ω*_*m*_ and *ω*_−*m*_ which are coupled by the intracavity population of *B*_0_. Their coupling leads to a pair of equations

$$\frac{{{{{{{{\rm{d}}}}}}}}}{{{{{{{{\rm{d}}}}}}}}t}{A}_{m} =-\frac{{\kappa }_{A,m}}{2}{A}_{m}-2i{g}_{0,-mm}{A}_{-m}^{* }{B}_{0}{e}^{-i{\delta }_{0,-mm}t}\\ \frac{{{{{{{{\rm{d}}}}}}}}}{{{{{{{{\rm{d}}}}}}}}t}{A}_{-m}^{* } =-\frac{{\kappa }_{A,-m}}{2}{A}_{-m}^{* }+2i{g}_{0,-mm}^{* }{A}_{m}{B}_{0}^{* }{e}^{i{\delta }_{0,-mm}t}$$

which become unstable for sufficiently large ∣*B*_0_∣. To see this note that *δ*_0,−*mm*_ = (Ω_0_ − 2*ω*_0_) − *ζ*_2_*m*^2^ allowing us to move into a rotating frame with

10 $${A}_{m}={\tilde{A}}_{m}\exp \left(-i\frac{{{\Delta }}+\mu -{\zeta }_{2}{m}^{2}}{2}t\right)$$

where *μ* ≡ Ω_0_ − 2*ω*_0_ is the *modal detuning* between the driven SH and closest FH mode, which in our experiment is set by tuning the temperature. In this frame, the equations become time-independent, and we obtain the stability criterion (assuming *κ*_±*m*_ are equal for simplicity):

11 $$16{|{g}_{0,-mm}|}^{2}{|{B}_{0}|}^{2}\ge {({{\Delta }}+\mu -{\zeta }_{2}{m}^{2})}^{2}+{({\kappa }_{A,m})}^{2}.$$

To relate this to the input photon flux at the SH frequency Ω_0_, we replace *B*_0_ using Eq. (9), to obtain

12 $$16{|{g}_{0,-mm}|}^{2}{|{G}_{0}|}^{2}{\kappa }_{B,0}^{{{{{{{{\rm{(e)}}}}}}}}}\ge ({{{\Delta }}}^{2}+{({\kappa }_{B,0}/2)}^{2})\times ({({{\Delta }}+\mu -{\zeta }_{2}{m}^{2})}^{2}+{({\kappa }_{A,m})}^{2}).$$

We can see from here that the lowest degenerate oscillation threshold can be achieved when *μ* = 0 and Δ = 0:

13 $$4{|{g}_{0,-mm}|}^{2}{|{G}_{0}|}^{2}{\kappa }_{B,0}^{{{{{{{{\rm{(e)}}}}}}}}}\ge {({\kappa }_{B,0}/2)}^{2}{({\kappa }_{A,m}/2)}^{2}\,{{{{{{{\rm{or}}}}}}}},$$

14 $${P}_{{{{{{{{\rm{th,0}}}}}}}}}=\frac{\hslash {{{\Omega }}}_{0}}{64{|{g}_{0,00}|}^{2}}\frac{{\kappa }_{B,0}^{2}{\kappa }_{A,0}^{2}}{{\kappa }_{B,0}^{{{{{{{{\rm{(e)}}}}}}}}}}.$$

More generally, the OPO will oscillate first in the mode *m* for which *P*_th,*m*_ is the lowest, where

15 $${P}_{{{{{{{{\rm{th}}}}}}}},m}=\frac{\hslash {{{\Omega }}}_{0}}{16{|{g}_{0,-mm}|}^{2}}\frac{1}{{\kappa }_{B,0}^{{{{{{{{\rm{(e)}}}}}}}}}}({{{\Delta }}}^{2}+{({\kappa }_{B,0}/2)}^{2})\times ({({{\Delta }}+\mu -{\zeta }_{2}{m}^{2})}^{2}+{({\kappa }_{A,m})}^{2}).$$

Here we’ve assumed again that the losses for the ±*m* modes are equal. Equation (15) shows that we can use the modal detuning *μ* and the driving detuning Δ to select which modes reach threshold first and oscillate as the power is increased. Assuming that *g*_0,−*mm*_ does not change significantly with the mode number, we see that for on-resonant driving Δ = 0, a minimum threshold can be achieved when *μ* = *ζ*_2_*m*^2^, as long as *μ* and *ζ*_2_ have the same sign. In our case, the waveguide has normal dispersion, so *ζ*_2_ is negative, and we have roughly *ζ*_2_/2*π* = −100 kHz. The relation $m\approx \sqrt{\mu /{\zeta }_{2}}$ shows that the mode number selected is very sensitive to the modal detuning (set by temperature) which makes the degenerate oscillation mode challenging to obtain in a system with a large resonator and therefore very small *ζ*_2_ mode-spacing dispersion parameter.

Interestingly, if the modal detuning *μ* is held constant while the pump detuning Δ is swept, the oscillation threshold can select very different modes *m* with only small changes in Δ. When the laser is nearly resonant with Ω_0_, so Δ is small compared to the *B* mode linewidth, the first term in parenthesis in Eq. (15) is minimized and does not vary strongly with detuning, while the second term is minimized whenever Δ + *μ* ≈ *ζ*_2_*m*^2^. This means that with a fixed laser input power, sweeping the laser across the second harmonic mode causes oscillation at very different mode numbers and explains the spectrum in Fig. 3b. For example, if we set Δ ≪ *κ*_*B*,0_, we would obtain an approximate equation for the oscillating mode index (which should be rounded to obtain an integer, and requires *μ* + Δ to have the same sign as *ζ*_2_):

16 $$m\approx \sqrt{\frac{\mu +{{\Delta }}}{{\zeta }_{2}}}$$

For the real device, we observe disorder in the loss rates for different signal modes, which can result from fabrication imperfections or coupler dispersion. We can account for that in our threshold calculation

17 $${P}_{{{{{{{{\rm{th}}}}}}}},m}=\frac{\hslash {{{\Omega }}}_{0}}{16{|{g}_{0,-mm}|}^{2}}\frac{1}{{\kappa }_{B,0}^{{{{{{{{\rm{(e)}}}}}}}}}}({{{\Delta }}}^{2}+{({\kappa }_{B,0}/2)}^{2})\times ({({{\Delta }}+\mu -{\zeta }_{2}{m}^{2})}^{2}+{\kappa }_{A,m}{\kappa }_{A,-m}).$$

To obtain a relation for the OPO power output, we solve Eqs. (6)–(7) for specific modes in steady-state. For the zero detuning of the pump mode Δ = 0 and assuming *μ* = *ζ*_2_*m*^2^, we have:

18 $${A}_{m}=\frac{4i{g}_{0,-mm}{A}_{-m}^{* }{B}_{0}}{{\kappa }_{A,m}}$$

19 $${B}_{0}=\frac{2i{g}_{0,-mm}^{* }{A}_{m}{A}_{-m}+\sqrt{{\kappa }_{B,0}^{{{{{{{{\rm{(e)}}}}}}}}}}{G}_{0}}{{\kappa }_{B,0}/2}.$$

Now, if we note that the oscillating amplitudes and coupling rate are complex ${A}_{m}=| {A}_{m}| \exp (i{\theta }_{m})$, ${g}_{k,00}=| {g}_{k,00}| \exp (i\varphi )$, we can substitute Eq. (18) to (19) and obtain

20 $$\frac{{\kappa }_{A,m}{\kappa }_{B,0}}{8i{|{g}_{0,-mm}|}^{2}}\frac{| {A}_{m}| }{| {A}_{-m}| }-2i| {A}_{m}| | {A}_{-m}| +\frac{\sqrt{{\kappa }_{B,0}^{{{{{{{{\rm{(e)}}}}}}}}}}}{| {g}_{0,-mm}| }{G}_{0}{e}^{i(\varphi -{\theta }_{m}-{\theta }_{-m})}=0.$$

This requires the exponential $\exp (i(\varphi -{\theta }_{m}-{\theta }_{-m}))$ to be purely imaginary, *φ* − *θ*_*m*_ − *θ*_−*m*_ = *π*/2 + *d* ⋅ *π*, where $d\in {\mathbb{Z}}$. This phase relation shows that the sum of the phases of the OPO output are locked to the phase of the pump. As a result, we can use Eq. (18) to find that

21 $$\frac{| {A}_{m}| }{| {A}_{-m}| }=\sqrt{\frac{{\kappa }_{A,-m}}{{\kappa }_{A,m}}},$$

and solve Eq. (20) for the photon flux of both signal modes of the OPO:

22 $${\left|{A}_{m}\right|}^{2}=\sqrt{\frac{{\kappa }_{A,-m}}{{\kappa }_{A,m}}}\frac{\sqrt{{\kappa }_{B,0}^{{{{{{{{\rm{(e)}}}}}}}}}}{G}_{0}}{2| {g}_{0,-mm}| }-\frac{{\kappa }_{B,0}{\kappa }_{A,m}}{16{|{g}_{0,-mm}|}^{2}}$$

23 $${\left|{A}_{-m}\right|}^{2}=\sqrt{\frac{{\kappa }_{A,m}}{{\kappa }_{A,-m}}}\frac{\sqrt{{\kappa }_{B,0}^{{{{{{{{\rm{(e)}}}}}}}}}}{G}_{0}}{2| {g}_{0,-mm}| }-\frac{{\kappa }_{B,0}{\kappa }_{A,-m}}{16{|{g}_{0,-mm}|}^{2}}.$$

To analyze the total output power of the OPO in experiment, we sum over the power of two signal modes

24 $${P}_{{{{{{{{\rm{out}}}}}}}}}=\frac{4{\eta }_{B,0}}{{{{\Omega }}}_{0}}({\eta }_{A,m}{\omega }_{m}+{\eta }_{A,-m}{\omega }_{-m})\times {P}_{{{{{{{{\rm{th,m}}}}}}}}}\left(\sqrt{\frac{{P}_{{{{{{{{\rm{B}}}}}}}},0}}{{P}_{{{{{{{{\rm{th,m}}}}}}}}}}}-1\right),$$

with ${\eta }_{k}={\kappa }_{k,0}^{{{{{{{{\rm{(e)}}}}}}}}}/{\kappa }_{k,0}$ for *k* = *A*, *B* being the cavity-waveguide coupling efficiency. *P*_B,0_ is the pump power of the SH mode and *P*_th,m_ is a generalized OPO threshold, which includes disorder in the total loss rates of fundamental modes:

25 $${P}_{{{{{{{{\rm{th,m}}}}}}}}}=\frac{\hslash {{{\Omega }}}_{0}}{64{|{g}_{0,-mm}|}^{2}}\frac{{\kappa }_{B,0}^{2}{\kappa }_{A,m}{\kappa }_{A,-m}}{{\kappa }_{B,0}^{{{{{{{{\rm{(e)}}}}}}}}}}=\frac{\hslash {{{\Omega }}}_{0}}{16{\eta }_{B}}\frac{\sqrt{{\kappa }_{A,m}{\kappa }_{A,-m}}}{{C}_{0,m}},$$

where ${C}_{0,m}\equiv 4| {g}_{0,-mm}{| }^{2}/(\sqrt{{\kappa }_{A,m}{\kappa }_{A,-m}}{\kappa }_{B,0})$ is the vacuum cooperativity for *m*^th^ pair of signal modes. Note that this relation agrees with Eq. (17) for the case of modal and laser detuning optimized for *m*^th^ OPO sideband.

### Second-harmonic generation efficiency

Starting from the coupled mode Eqs. (6) and (7), we now assume that only *A*_0_ is excited, i.e., we are driving the mode at *ω*_0_ and all other mode FH amplitudes are 0:

26 $$\frac{{{{{{{{\rm{d}}}}}}}}}{{{{{{{{\rm{d}}}}}}}}t}{A}_{0}=-\frac{{\kappa }_{A,0}}{2}{A}_{0}-2i\mathop{\sum}\limits_{k}{g}_{k,00}{A}_{0}^{* }{B}_{k}{e}^{-i{\delta }_{k,00}t}-\sqrt{{\kappa }_{A,0}^{{{{{{{{\rm{(e)}}}}}}}}}}{F}_{0}{e}^{-i({\omega }_{L}-{\omega }_{0})t}$$

27 $$\frac{{{{{{{{\rm{d}}}}}}}}}{{{{{{{{\rm{d}}}}}}}}t}{B}_{k}=-\frac{{\kappa }_{B,k}}{2}{B}_{k}-i{g}_{k,00}^{* }{A}_{0}^{2}{e}^{+i{\delta }_{k,00}t}.$$

To solve these equations, we go into a frame that rotates with the laser detuning frequency *δ* = *ω*_*L*_ − *ω*_0_, so ${A}_{0}={\tilde{A}}_{0}{e}^{-i\delta t}$, ${B}_{k}={\tilde{B}}_{k}{e}^{-i(2\delta -{\delta }_{k,00})t}={\tilde{B}}_{k}{e}^{-i(2{\omega }_{L}-{{{\Omega }}}_{k})t}$:

28 $$\frac{{{{{{{{\rm{d}}}}}}}}}{{{{{{{{\rm{d}}}}}}}}t}{\tilde{A}}_{0}=\left(i\delta -\frac{{\kappa }_{A,0}}{2}\right){\tilde{A}}_{0}-2i\mathop{\sum}\limits_{k}{g}_{k,00}{\tilde{A}}_{0}^{* }{\tilde{B}}_{k}-\sqrt{{\kappa }_{A,0}^{{{{{{{{\rm{(e)}}}}}}}}}}{F}_{0}$$

29 $$\frac{{{{{{{{\rm{d}}}}}}}}}{{{{{{{{\rm{d}}}}}}}}t}{\tilde{B}}_{k}=\left(i(2{\omega }_{L}-{{{\Omega }}}_{k})-\frac{{\kappa }_{B,k}}{2}\right){\tilde{B}}_{k}-i{g}_{k,00}^{* }{\tilde{A}}_{0}^{2}.$$

We can solve these in steady state to obtain:

30 $${\tilde{B}}_{k}=\frac{i{g}_{k,00}^{* }{\tilde{A}}_{0}^{2}}{i(2{\omega }_{L}-{{{\Omega }}}_{k})-\frac{{\kappa }_{B,k}}{2}}$$

31 $$0=\left(i\delta -\frac{{\kappa }_{A,0}}{2}\right){\tilde{A}}_{0}+\mathop{\sum}\limits_{k}\frac{2{|{g}_{k,00}|}^{2}{|{\tilde{A}}_{0}|}^{2}}{i(2{\omega }_{L}-{{{\Omega }}}_{k})-\frac{{\kappa }_{B,k}}{2}}{\tilde{A}}_{0}-\sqrt{{\kappa }_{A,0}^{{{{{{{{\rm{(e)}}}}}}}}}}{F}_{0}$$

There are a couple of interesting things to note about the last equation. Note that each SH mode at Ω_*k*_ contributes effective nonlinear loss and detuning terms to the FH mode at *ω*_0_:

32 $${{{{{{{\rm{Detuning}}}}}}}}{:}\,\,\,-\mathop{\sum}\limits_{k}\frac{2(2{\omega }_{L}-{{{\Omega }}}_{k})}{{(2{\omega }_{L}-{{{\Omega }}}_{k})}^{2}+{\left(\frac{{\kappa }_{B,k}}{2}\right)}^{2}}{g}_{k,00}^{2}{\left|{A}_{0}\right|}^{2}$$

33 $${{{{{{{\rm{Loss}}}}}}}}{:}\,\,\,\mathop{\sum}\limits_{k}\frac{2{\kappa }_{B,k}}{{(2{\omega }_{L}-{{{\Omega }}}_{k})}^{2}+{\left(\frac{{\kappa }_{B,k}}{2}\right)}^{2}}{g}_{k,00}^{2}{\left|{A}_{0}\right|}^{2}$$

For large 2*ω*_*L*_ − Ω_*k*_ ≫ *κ*_*b*_, we see an effect which is primarily a frequency shift and looks much like a *χ*^(3)^ cavity frequency shift.

From here on, we assume that only one SH mode (*k* = 0) is significantly excited. The photons generated at the *B*_0_ mode frequency are emitted from the device generating a photon flux ∣*G*_out,0_∣^2^ at the SH frequency where ${G}_{{{{{{{{\rm{out}}}}}}}},0}=\sqrt{{\kappa }_{B,0}^{{{{{{{{\rm{(e)}}}}}}}}}}{\tilde{B}}_{0}$. To find ${\tilde{B}}_{0}$, we need to calculate ${\tilde{A}}_{0}$ (Eq. (30)), which is given implicitly by

34 $$0=\left(i\delta -\frac{{\kappa }_{A,0}}{2}\right){\tilde{A}}_{0}+\frac{2{|{g}_{0,00}|}^{2}{\left|{\tilde{A}}_{0}\right|}^{2}}{i(2{\omega }_{L}-{{{\Omega }}}_{0})-\frac{{\kappa }_{B,0}}{2}}{\tilde{A}}_{0}-\sqrt{{\kappa }_{A,0}^{{{{{{{{\rm{(e)}}}}}}}}}}{F}_{0}.$$

For the fits shown in the paper, this equation was solved numerically. Here we assume *δ* = 0 and approximate the solutions in two limits, (1) the low-power limit where the first term is dominant, and (2) the high-power limit where the second term is dominant. The cross-over between these two limits occurs at

35 $$2{C}_{0}{n}_{A}^{(0)}=1$$

where *C*_0_ = 4∣*g*_0,00_∣^2^/*κ*_*A*,0_*κ*_*B*,0_ is a cooperativity parameter and ${n}_{A}^{(0)}=4{\kappa }_{A,0}^{{{{{{{{\rm{(e)}}}}}}}}}| {F}_{0}{| }^{2}/{\kappa }_{A,0}^{2}$ is the number of intracavity photons which would be excited in the absence of nonlinearity. Solving the above equation in the two limits gives us

$${\left|{\tilde{A}}_{0}\right|}^{2}={n}_{A}^{(0)},{{{{{{{\rm{and}}}}}}}}\,\,\,{\left|{\tilde{A}}_{0}\right|}^{2}={\left(\frac{{n}_{A}^{(0)}}{4{C}_{0}^{2}}\right)}^{1/3}$$

for the low- and high-power limits, respectively. We define the second harmonic generation power efficiency

36 $${\eta }_{{{{{{{{\rm{SHG}}}}}}}}}\equiv \frac{{P}_{{{{{{{{\rm{out}}}}}}}}}}{{P}_{{{{{{{{\rm{in}}}}}}}}}}=\frac{2{\left|{G}_{{{{{{{{\rm{out}}}}}}}},0}\right|}^{2}}{{\left|{F}_{0}\right|}^{2}},$$

which after some manipulation, can be written in terms of $| {\tilde{A}}_{0}{| }^{2}$:

37 $${\eta }_{{{{{{{{\rm{SHG}}}}}}}}}	=\; \frac{8{\eta }_{A}{\eta }_{B}{C}_{0}}{{n}_{A}^{(0)}}{\left|{\tilde{A}}_{0}\right|}^{4}\\ 	= \left\{\begin{array}{ll}8{\eta }_{A}{\eta }_{B}{C}_{0}{n}_{A}^{(0)} &{{{{{{{\rm{low}}}}}}}}\,{{{{{{{\rm{power}}}}}}}}\\ \frac{4{\eta }_{A}{\eta }_{B}}{{\left(2{n}_{A}^{(0)}{C}_{0}\right)}^{1/3}} &{{{{{{{\rm{high}}}}}}}}\,{{{{{{{\rm{power}}}}}}}}\end{array}\right.$$

It is apparent that at low power, the efficiency increases linearly, but is then saturated at high power. This can be understood from an impedance matching perspective. As the pump power is increased, the FH cavity resonance senses a two-photon loss proportional to $8| {g}_{0,00}{\tilde{A}}_{0}{| }^{2}/{\kappa }_{B,0}$ (see Eq. (33)). As this loss starts to exceed the cavity linewidth, its effective coupling rate to the waveguide is reduced, preventing input light from coupling efficiently into the cavity to be frequency-doubled. Designing an overcoupled resonator can compensate for the nonlinear loss rate and allow for higher maximum efficiencies compared to critically coupled or undercoupled resonators, at the expense of increased OPO threshold power. At very high power, the efficiency actually begins to go down as *P*^−1/3^. The model assumes that only the ${\tilde{B}}_{0}$ and ${\tilde{A}}_{0}$ modes are excited. As we saw in the case of a directly driven OPO, at sufficiently large ${\tilde{B}}_{0}$, ${\tilde{A}}_{\pm m}$ start to oscillate, which causes this model to break down and the system to go into cascaded optical parametric oscillation.

### Cascaded optical parametric oscillation

Consider the same driving as in the previous section, where a laser drive at the fundamental with frequency *ω*_*L*_ excites ${\tilde{A}}_{0}$ and generates an intracavity population in the second harmonic mode ${\tilde{B}}_{0}$. From the section on the oscillation threshold, we know that at a sufficiently value of $| {\tilde{B}}_{0}|$, the equations of motion for mode amplitudes ${\tilde{A}}_{\pm m}$ become unstable and set of oscillations, with a threshold condition given by an equation very similar to Eq. (11):

$$16{|{g}_{0,-mm}|}^{2}{\left|{\tilde{B}}_{0}\right|}^{2}\ge {\left(2\delta +\mu -{\zeta }_{2}{m}^{2}\right)}^{2}+{\kappa }_{A,m}{\kappa }_{A,-m}.$$

We call this a cascaded OPO, since a cascade of two back-to-back *χ*^(2)^ processes lead to parametric oscillation. It is clear from the oscillation condition that the threshold is highly detuning-dependent, and also depends on the dispersion parameter *ζ*_2_. In our case, *ζ*_2_/2*π* ≈ −100 kHz and so the oscillation threshold is lower with the laser tuned to the red side (*δ* < 0) when the modal detuning *μ* ≈ 0.

### Nonlinear coupling rate

We derive the nonlinear coupling rate from the interaction energy density in the three-wave mixing process. Given the electric field distribution **E** = (*E*^*x*^, *E*^*y*^, *E*^*z*^) The interaction energy density is given by:

38 $${U}_{{\chi }^{(2)}}=\frac{{\varepsilon }_{0}}{3}\mathop{\sum}\limits_{\alpha \beta \gamma }{\chi }_{\alpha \beta \gamma }^{(2)}{E}^{\alpha }{E}^{\beta }{E}^{\gamma },$$

each of the three waves can be expressed using spatial complex amplitudes **E**_*m*_, **E**_*n*_, **E**_*k*_ as follows:

39 $${{{{{{{\bf{E}}}}}}}}={A}_{m}{{{{{{{{\bf{E}}}}}}}}}_{m}{e}^{-i{\omega }_{m}t}+{A}_{n}{{{{{{{{\bf{E}}}}}}}}}_{n}{e}^{-i{\omega }_{n}t}+{B}_{k}{{{{{{{{\bf{E}}}}}}}}}_{k}{e}^{-i{{{\Omega }}}_{k}t}+{{{{{{{\rm{h.c.}}}}}}}}$$

To calculate the nonlinear coupling rate we focus on three specific modes in the sum and evaluate Eq. (38) by averaging away the rapidly rotating terms. It selects only energy-conserving terms of the sum. Since the second-order nonlinear tensor has a full permutation symmetry, for the non-degenerate we find that

40 $${U}_{{\chi }^{(2)}}^{k,nm}=2{\varepsilon }_{0}\mathop{\sum}\limits_{\alpha \beta \gamma }{\chi }_{\alpha \beta \gamma }^{(2)}\left({E}_{k}^{\alpha * }{E}_{m}^{\beta }{E}_{n}^{\gamma }{B}_{k}^{* }{A}_{m}{A}_{n}+{{{{{{{\rm{h.c.}}}}}}}}\right)=2{\varepsilon }_{0}({{{{{{{{{\bf{E}}}}}}}}}_{k}}^{* }{\bar{\bar{\chi }}}^{(2)}:{{{{{{{{\bf{E}}}}}}}}}_{m}{{{{{{{{\bf{E}}}}}}}}}_{n}{B}_{k}^{* }{A}_{m}{A}_{n}+{{{{{{{\rm{h.c.}}}}}}}}).$$

Integrating over this energy density gives us the total energy of the system, which we use to derive the equations of motion (6) and (7). We choose normalization of the modal field **E**_*k*_ so that the total energy corresponding to an amplitude *A*_*k*_ is *ℏω*_*k*_∣*A*_*k*_∣^2^. More precisely, given unitless field profiles **e**_*i*_ (with $\max ({{{{{{{{\bf{e}}}}}}}}}_{i})=1$), we introduce normalization factors *N*_*i*_, defined by **E**_*i*_ = *N*_*i*_**e**_*i*_. The energy condition then fixes these normalization factors as

41 $${N}_{i}	=\; \sqrt{\frac{\hslash {\omega }_{i}}{2\int {{{{{{{{{\bf{e}}}}}}}}}_{i}}^{* }\bar{\bar{\varepsilon }}({{{{{{{\bf{r}}}}}}}}){{{{{{{{\bf{e}}}}}}}}}_{i}dV}}\\ 	=\; \sqrt{\frac{\hslash {\omega }_{i}}{2{\varepsilon }_{0}L\int {{{{{{{{{\bf{e}}}}}}}}}_{i}}^{* }{\bar{\bar{\varepsilon }}}_{r}({{{{{{{\bf{r}}}}}}}}){{{{{{{{\bf{e}}}}}}}}}_{i}dA}}\\ 	=\; \sqrt{\frac{\hslash {\omega }_{i}}{2{\varepsilon }_{0}L{\bar{n}}_{i}^{2}}}\frac{1}{\sqrt{{{{{{{{{\mathcal{A}}}}}}}}}_{i}}}$$

Here, we introduced the effective mode area for each mode as ${{{{{{{{\mathcal{A}}}}}}}}}_{i}={\int}_{A}| {{{{{{{\bf{e}}}}}}}}_i{| }^{2}dA$, and define the average index as ${\bar{n}}_{i}^{2}=\int {{{{{{{{{\bf{e}}}}}}}}}_{i}}^{* }{\bar{\bar{\varepsilon }}}_{r}({{{{{{{\bf{r}}}}}}}}){{{{{{{{\bf{e}}}}}}}}}_{i}dA/{{{{{{{{\mathcal{A}}}}}}}}}_{i}$. To find the energy, we integrate Eq. (40) over the mode volume. We account for a partially-poled racetrack resonator by introducing the poled length fraction *λ* as a ratio of the poled region to the total resonator length *L*. The final expression for the nonlinear coupling rate is given by:

42 $${g}_{k,nm}=\frac{\lambda }{\sqrt{2}\pi }\sqrt{\frac{\hslash {\omega }_{m}{\omega }_{n}{{{\Omega }}}_{k}}{{\varepsilon }_{0}L{\bar{n}}_{k}^{2}{\bar{n}}_{m}^{2}{\bar{n}}_{n}^{2}}}\frac{{{{{{{{\mathcal{O}}}}}}}}}{\sqrt{{{{{{{{{\mathcal{A}}}}}}}}}_{m}{{{{{{{{\mathcal{A}}}}}}}}}_{n}{{{{{{{{\mathcal{A}}}}}}}}}_{k}}},$$

where ${{{{{{{\mathcal{O}}}}}}}}$ represents the mode overlap integral over the waveguide cross-section area

43 $${{{{{{{\mathcal{O}}}}}}}}={\int}_{A}{{{{{{{{{\bf{e}}}}}}}}}_{k}}^{* }{\bar{\bar{\chi }}}^{(2)}:{{{{{{{{\bf{e}}}}}}}}}_{m}{{{{{{{{\bf{e}}}}}}}}}_{n}dA.$$

For our numerical waveguide calculations we use a finite-element mode solver (COMSOL).

### Numerical simulations of dynamics

**Fig. 6:**
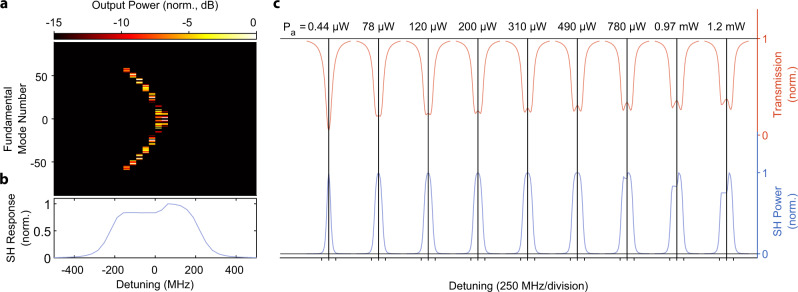
Simulated effect of 0.1% disorder in the mode-to-mode loss rates on the cascaded OPO. **a** Cascaded OPO signal as a function of pump detuning, simulated at 0.97 mW of pump power. **b** Second Harmonic lineshape corresponding to the cascaded OPO in panel (**a**). **c** Lineshape evolution as a function of power, asymmetry is developed below 780 μW, which agrees with the experiment.

**Fig. 7:**
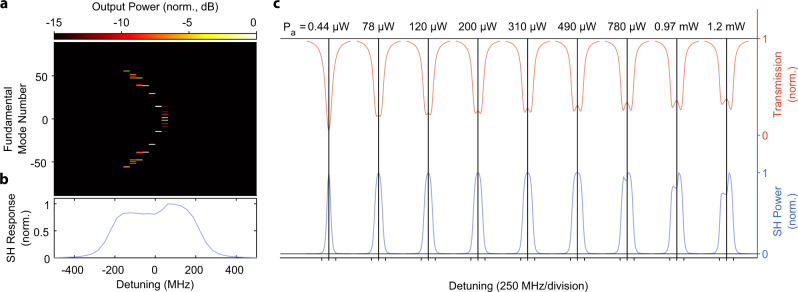
Simulated effect of 1% disorder in the mode-to-mode loss rates on the cascaded OPO. **a** Cascaded OPO signal as a function of pump detuning, simulated at 0.97 mW of pump power. **b** Second Harmonic lineshape corresponding to the cascaded OPO in panel (**a**). **c** Lineshape evolution as a function of power, asymmetry is developed below 780 μW, which agrees with the experiment.

**Fig. 8:**
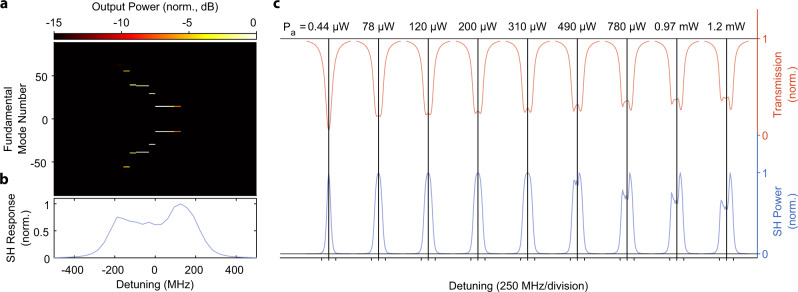
Simulated effect of 10% disorder in the mode-to-mode loss rates on the cascaded OPO. **a** Cascaded OPO signal as a function of pump detuning, simulated at 0.97 mW of pump power. **b** Second Harmonic lineshape corresponding to the cascaded OPO in panel (**a**). **c** Lineshape evolution as a function of power, asymmetry is developed around 490 μW, lower than in the experiment.

We numerically integrate the coupled-mode differential Eqs. (6) and (7) to understand how the transmission spectra change when the system starts to oscillate and how disorder affects the emission spectra of the cascaded OPO. We integrate the coupled-mode equations with 181 *A* modes and 31 *B* modes for 600 ns which is sufficiently long for the system to stabilize. We use the measured parameters from the SHG experiment for the *ω*_0_ and Ω_0_ modes, and assume that the other modes are spaced by the measured FSR (which agrees with the theory prediction) and have the same quality factors. The resulting spectra for are shown in Fig. 6. We then perform the same simulation but with the quality factors and detunings of the other modes now having disorder (normally distributed fluctuations of total *Q* and mode frequency) on the order of 1% (Fig. 7) and 10% (Fig. 8) of the cavity linewidth.

## Data Availability

The data sets generated during and/or analyzed during this study are available from the corresponding authors on request.
